# Challenges of alcohol use during pregnancy: maternal and fetal implications

**DOI:** 10.1192/j.eurpsy.2025.2405

**Published:** 2025-08-26

**Authors:** M. M. Figueiredo, A. Falcão, V. Nogueira, J. Teixeira

**Affiliations:** 1 Centro Hospitalar Setúbal, ULS Arrábida, Setúbal; 2 Centro Hospitalar Psiquiátrico de Lisboa, Lisboa, Lisboa, Portugal; 3 Serviço de Alcoologia e Novas Dependências, Centro Hospitalar Psiquiátrico de Lisboa, Lisboa, Lisboa, Portugal

## Abstract

**Introduction:**

In recent years, there has been an increase in alcohol consumption among women. Given that a significant percentage (1 in 3) of pregnancies are unplanned, fetal exposure to alcohol is inevitable in some.

**Objectives:**

The main objective of this work is to present a review of the current state of the art regarding the impact of alcohol use during pregnancy.

**Methods:**

Evidence-based review, using a search on PubMed and selection of the most relevant studies on this topic, published in the last decade.

**Results:**

Although the international consensus recommends total abstinence from alcohol consumption during pregnancy, its global prevalence is 9,8%, with the highest percentage found in Europe (25,2%), which constitutes an important public health problem. Contrary to expectations, a recent study highlights that women who continue to consume alcohol during pregnancy were more likely to be older and have a higher socioeconomic status and educational level. Alcohol consumption during pregnancy is associated with high rates of maternal and child morbidity and mortality, with serious social, personal and family consequences. Prenatal alcohol exposure is the leading preventable cause of birth defects and neurodevelopmental disorders. The psychopharmacological treatment of alcohol use disorder during pregnancy is a challenge, given the limited evidence.

**Conclusions:**

The main guidelines recommend total alcohol abstinence as no safe amount to consume has been identified. Stopping alcohol consumption during pregnancy is the only effective way to eliminate the risk of complications associated with this substance. A focus on early prevention and identification is essential, as well as, when necessary, referral for specialized treatment.

**Disclosure of Interest:**

None Declared

